# Fixation in slipped capital femoral epiphysis avoiding femoral-acetabular impingement

**DOI:** 10.1186/s13018-017-0663-3

**Published:** 2017-10-30

**Authors:** Francesco Falciglia, Angelo G. Aulisa, Marco Giordano, Vincenzo Guzzanti

**Affiliations:** 1 Department of Orthopaedics and Traumatology, Institute of Scientific Research, Children’s Hospital Bambino Gesù, P.zza S. Onofrio 4, 00165 Rome, Italy; 2 University of Cassino, Strada Folcare, 4, 03043 Cassino, FR Italy

**Keywords:** Slipped capital femoral epiphysis, Treatment, Stabilization, Remodeling, Impingement

## Abstract

**Background:**

The appropriate treatment in mild slipped capital femoral epiphysis (SCFE) should not only prevent further slipping of the epiphysis but also address potential femoroacetabular impingement by restoring the anatomy of the proximal femur. The aim of this study was to quantify length of the remodeling phase mediated by growth of the femoral neck, after treatment of SCFE with a screw designed to prevent premature closure of the physis and provide stability.

**Methods:**

Between 2001 and 2011, 38 patients with unilateral mild SCFE were treated by fixation in situ using a modified screw which does not cause premature physeal arrest. Twenty-four patients were investigated for clinical and radiological evidence of femoroacetabular impingement immediately after surgery, at 6- and 12-month follow-ups. Statistical analysis was performed comparing measurements of neck length and the α angle of the affected and contralateral side.

**Results:**

Mean α angle immediately after pinning was 56.2 ± 10.6° on the anteroposterior view and 91.4 ± 8.2° on the lateral view. These measurements significantly improved at 6 months post-op to 48.9 ± 5.4° on the anteroposterior view and 51.2 ± 6.5° on the lateral view (*p* < 0.0001). At 12 months from surgery, AP view α angle was 43.0 ± 2.8° (*p* < 0.0001) and lateral view was 44.2 ± 4.1° (*p* < 0.0001). We observed a similar growth rate and speed of the femoral neck of both the affected and unaffected sides during the first year of treatment. The clinical results in all patients were rated as excellent.

**Conclusion:**

Our data supports the use of a surgical technique that allows residual growth of the femoral neck following mild SCFE and permits restoration of the anatomy of the proximal femur while avoiding development of femoroacetabular impingement following mild SCFE.

## Background

Slipped capital femoral epiphysis (SCFE) is a possible cause of femoro-acetabular impingement (FAI) and subsequent premature osteoarthritis of the hip [1–3]. Surgical treatment of SCFE depends on the severity of the slip. For mild slips, with angulation < 30°, fixation of the epiphysis in situ by smooth pins or screws is recommended [4–7]. Ward et al. (1992) [8] found that the more central the fixation, the earlier the physeodesis, especially using trans-physeal screw stabilization. Two studies suggest that after slippage of the epiphysis, changes occur at the acetabular labrum and adjacent cartilage which act as precursors of osteoarthritis of the affected hip joint [9, 10].

These two studies, which also analyzed moderate and severe SCFE, demonstrated articular erosions and scars and/or tears of the labrum, with damage to the articular cartilage ranging from superficial abrasions to full-thickness loss caused by a prominent femoral metaphysis protruding beyond or at least level with the physis.

Due to such findings, these studies recommended an osteochondroplasty at the femoral head-neck junction in addition to stabilization of the epiphysis in situ, in an attempt to reduce risk of developing secondary osteoarthritis [11].

As described by Menelaus (1991) [12], the femoral neck grows at an estimated rate of 4.0 mm/year. In younger patients, premature closure of the physis risks development of a short femoral neck, producing a short lever arm and a high-riding trochanter with progressive deformity of the epiphyseal–metaphyseal complex of the proximal femur, leading to premature osteoarthritis [13, 14]. A standard screw fixation associated with an osteochondroplasty may then cause a premature closure of the physis and consequent deformity of the head-neck junction associated with an overgrowth of the great trochanter. This might lead to intra- and extra-articular impingements [14], making the additional osteochondroplasty useless.

Our hypothesis is then to avoid an osteochondroplasty trusting in a biological remodeling due to growth. To reach this target, an ideal method in SCFE should stabilize the epiphysis without causing unwanted premature closure of the physis. Histologic, ultrastructural, and clinical studies post stabilization reported remodeling with normalization of the physis [15–21]. Remodeling due to the growth of the neck can reduce or eliminate head/neck incongruities.

Guzzanti et al. (2004) [19] reported the results obtained with a modified screw to stabilize the slip in younger patients with SCFE. This method avoided leg-length discrepancy and maintained articular trochanteric distance and growth of the epiphyseal–metaphyseal complex. Since some authors [11] recommended osteochondroplasty in addition to traditional in situ fixation to avoid the progression of the labrum and articular lesions, we decided to review the results provided by our method.

The aim of this study was to evaluate the remodeling speed of the deformity in all the patients with monolateral mild SCFE treated by in situ fixation using Guzzanti’s modified screw [19].

## Methods

Ethical approval was obtained from the Ethics Committee of the Ospedale Pediatrico Bambino Gesù in Rome, Italy. Informed consent was obtained from all patients examined. Clinical and radiological records of all patients treated for a SCFE between 2001 and 2011 were reviewed.

Inclusion criteria for this study were as follows: (1) Patients with unilateral stable or unstable SCFE with posterior slippage < 35°. We accepted 5° greater than 30° to account for the possibility of error when measuring. (2) Surgical treatment using in situ fixation with a modified cannulated screw [19]. (3) Complete clinical and radiological records for review from presentation to at least 1-year follow-up. Data was collected immediately post-surgery, at 6 months and at 1 year.

Exclusion criteria included hip trauma during the follow-up period, associated metabolic or endocrine disease, involvement of the contralateral hip during follow-up, and/or incomplete medical and imaging records. Medical charts and radiographs were reviewed to determine age at presentation, sex, laterality, postoperative complications, and outpatient follow-ups.

### Operative technique

Surgery was carried out under fluoroscopic control. Spontaneous reduction occurred in the unstable slips when positioning the patient. A modified cannulated screw 90–120-mm long was used. The modified AO cannulated screw (HIT-MEDICA, Rimini, Italy) had a distal segment, with the original six threads reduced to three and were 9-mm long and 6.4 mm in diameter. The screw was inserted over a guide wire after pre-drilling. The threaded portion of the screw was placed entirely within the epiphysis with the smooth shank crossing the physis, femoral neck, and lateral femoral cortex. The screw head was allowed to remain about 1.5–2 cm to the side of the lateral femoral cortex (always under the iliotibial band to avoid disturbance to the patients) to provide for neck growth. This distance was based on the predicted growth of the proximal femur, plus a few additional millimeters, to facilitate removal using a guide wire if necessary. The position of the screw in the central quadrant of the femoral epiphysis was confirmed by multiple fluoroscopic views. The screw tip was positioned more than 2.5 mm from the epiphyseal subchondral bone. After insertion of the screw, the hip was taken through a full range of movement with multiple fluoroscopic views to check the final hardware position. Post-operation, patients with a stable SCFE were allowed to weight-bear as tolerated. Those with an unstable SCFE used crutches with partial weight-bearing for 4 weeks.

Pre-operative assessment included clinical and radiological classification of the SCFE in terms of severity and type (stable/unstable). The pre- and post-operative head-neck slip angles were measured on anteroposterior (AP) and lateral radiographs (with the leg held in a device at 90° of flexion and 45° of abduction. This is a modified abduction of the 90° Dunn view position [22]) taken at a standard 100-cm beam distance [23]. Any change of 5° or more was considered significant. A negative value was considered a decrease and a positive value an increase in slip angle. Radiographic measurements were made digitally using the CARESTREAM DRX-Evolution system. Slip severity was measured on pre-operative lateral radiographs with the posterior sloping angle (PSA) [23].

**Fig. 1:**
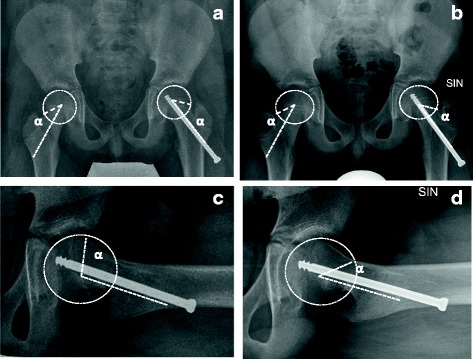
SCFE of the left hip in an 11.2-year-old boy. **a**, **b** AP view radiograms: **a** post-operative α angle and **b** α angle at 12 months FU. **c**, **d** Lateral view radiograms: **c** post-operative α angle and **d** α angle at 12 months FU: observe the normalization of the angle and the absence of head/neck deformity (case 23 in Tables 1 and 2)

PSA is the angle between the plane of the physis and the line perpendicular to the femoral neck axis. To assess cam deformity, two measurements were performed: alpha (α) angle in AP and lateral view radiographs in the affected and unaffected hips (Fig. 1).

**Fig. 2:**
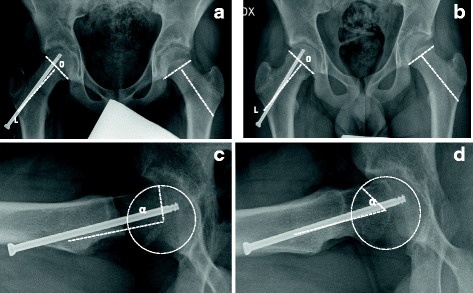
SCFE of the right hip in a 13.7-year-old boy. **a**, **b** AP view radiograms: **a** post-operative and **b** at 12 months FU: neck length (O-L) was measured from the lateral femoral cortex drawing a line perpendicular to the physis plane; the neck grew in the same way on the affected and unaffected sides, as growth of the femoral neck progressed, the screw advanced into the femoral neck from the lateral femoral cortex closing the gap between the lateral femoral cortex and the screw head. **c**, **d** Lateral view radiograms: **c** post-operative α angle and **d** α angle at 12 months FU: observe the normalization of the angle and the absence of head/neck deformity. Remodeling, due to the growth, eliminated the bump of the neck evident in the postoperative radiogram (**c**) (case 16 in Tables 1 and 2)

Measurements were performed immediately after pinning, at 6 months and at 12 months post-operatively. The α angle was measured on AP and lateral view radiograms following the methodology of Clohisy et al. (2008) [22]. It is calculated by measuring the angle between two lines: (1) a line drawn connecting the center of the femoral head to the point on the anterolateral and lateral view aspect of the head-neck junction, where the radius of the femoral head is found in the acetabulum (i.e., where a prominence starts), and (2) a line drawn through the center of the femoral neck, connecting to the center of the femoral head. An α angle greater than 50° is suggestive of cam deformity [24]. Concurrently, neck length was measured on a line drawn from the lateral femoral cortex to the physis on the AP view, as shown in Fig. 2.

Post-operation and at the 12-month follow-up, limb length discrepancy was clinically measured (from anterior iliac superior spine to tibial malleolus) and differences between the affected and unaffected sides noted.

All measurements were independently determined by two observers (FF and AGA), and inter-observer correlations were evaluated by chi-square test.

### Outcome criteria

Clinical follow-up assessment of the hip was made using the modification by Zahrawi et al. (1983) [25] of the classification of Heyman and Herndon (1954) [26] in which an excellent result has a normal range of hip movement, no limp, no pain, and leg shortening of < 1 cm. A good result had slight limitation of internal rotation, occasional pain, and leg shortening of < 1 cm. A fair result had persistent mild pain, loss of internal rotation, abduction, and leg shortening of > 1 cm. A poor result did not meet any of the above criteria. (We preferred this classification to the Harris Hip Score because it included leg shortening. We believe this is one parameter that must be considered and compared with traditional screw fixation in other papers.)

### Statistics

Descriptive statistics were used for the variables of our cohort. The paired Student’s *t* test was used to analyze the differences between a normal hip and a pathologic hip when the data was parametric, and the Pearson correlation was used to correlate the results with the collected data. Outcome data for each group are presented as the mean and standard deviation. The mean difference between groups and a 95% confidence interval are provided. A *p* value of < 0.05 was considered significant. Statistical analysis was performed using GraphPad Prism 5 computer software.

## Results

Between 2000 and 2011, we treated 38 patients with a unilateral mild SCFE by fixation in situ using Guzzanti’s method [19]. Twenty-four patients, 7 females and 17 males, with a mean age of 12.7 years (min 11–max 14.8) met the inclusion criteria (Table 1).

**Table 1:** Clinical and radiological data

Case	Age	Gender	Side	Type	PSA	αAPn	αAPp post-op	αAPp FU-6m	αAPp FU-12m	αLn	αLp post-op	αAPp FU-6m	αLp FU-12m
1	12.5	F	Left	Stable	34°	38°	70°	50°	42°	38°	90°	55°	44°
2	12.8	F	Left	Stable	34°	40°	58°	48°	46°	41°	94°	46°	42°
3	13.2	M	Right	Stable	22°	43°	84°	54°	45°	39°	60°	46°	44°
4	12.5	M	Right	Stable	26°	41°	80°	49°	46°	42°	92°	53°	48°
5	12.2	F	Right	Unstable	28°	43°	50°	52°	40°	42°	95°	48°	42°
6	12.1	F	Left	Stable	34°	40°	56°	46°	38°	42°	85°	43°	39°
7	14.1	M	Left	Stable	26°	43°	50°	44°	44°	44°	90°	50°	46°
8	13.9	M	Right	Stable	30°	43°	50°	48°	45°	42°	95°	55°	48°
9	14.6	M	Left	Stable	34°	42°	59°	55°	49°	38°	90°	60°	45°
10	12.2	F	Right	Stable	22°	42°	52°	52°	42°	38°	90°	55°	45°
11	11.9	F	Left	Stable	28°	42°	45°	42°	43°	44°	96°	45°	40°
12	13.3	M	Left	Stable	30°	40°	57°	54°	40°	42°	85°	51°	45°
13	12.8	F	Right	Stable	34°	45°	57°	51°	48°	42°	96°	62°	45°
14	12.9	M	Left	Stable	22°	45°	48°	47°	45°	41°	88°	41°	40°
15	14.4	M	Left	Stable	30°	42°	58°	47°	44°	42°	100°	42°	40°
16	13.8	M	Right	Stable	32°	43°	70°	65°	40°	44°	100°	45°	42°
17	11.8	M	Left	Stable	20°	44°	40°	40°	40°	42°	92°	50°	42°
18	13.5	M	Right	Unstable	28°	43°	59°	46°	42°	44°	90°	48°	44°
19	12.7	M	Left	Unstable	20°	43°	49°	45°	45°	44°	100°	60°	44°
20	12.0	M	Right	Stable	32°	42°	56°	54°	40°	38°	84°	54°	45°
21	12.7	M	Right	Stable	22°	42°	45°	42°	42°	38°	100°	65°	44°
22	12.5	M	Right	Stable	26°	40°	50°	45°	44°	42°	90°	51°	46°
23	11.3	M	Left	Stable	34°	42°	56°	52°	40°	38°	98°	58°	44°
24	11.8	M	Left	Unstable	30°	44°	50°	46°	42°	42°	94°	48°	44°

Clinical and radiological data: *PSA* posterior slip angle, *α* alpha angle, *AP* anteroposterior view radiogram, *n* normal unaffected side, *L* lateral view radiogram, *P* pathologic affected side, *FU* follow-up, *m* months

There were 20 stable and 4 unstable hips. No SCFE was noted on the contralateral side in this group during the 12 months post operation. There was no evidence of avascular necrosis or chondrolysis. There were no cases of hardware failure. There was no evidence of slip progression in any patient. The time from presentation to surgery for those with an unstable slip was from 12 to 24 h. Spontaneous reduction of the slip in all 4 unstable cases followed positioning the patient with the hip in extension and neutral rotation.

All measurements were independently determined by two observers (FF and AGA). Inter-observer correlations (using chi-square tests) were evaluated and no significant statistical differences were observed. The data tables reported are the mean value between the two observers.

The mean α angle immediately after pinning on the AP view was 56.2 ± 10.6° and on the lateral view 91.4 ± 8.2°.

At the 6-month follow-up, the α angle of the affected hip compared with the control (contralateral unaffected hip) was statistically significantly different with a *p* = 0.0001. The mean differences of 6° on the AP and 10° on lateral radiograms had an improvement of about 10° and 40°, respectively (Table 1).

At 12 months, the α angle compared with control was statistically significantly different (*p* = 0.002), the difference being 2.5° on the lateral radiogram.

On AP radiograms, there was no statistically significant difference observed between the involved and uninvolved sides (*p* = 0.1). (Table 1) (Figs. 1 and 2).

No closure of the growth plate was observed at 12 months in these cases.

A limb length discrepancy of ≤ 1 cm (the affected limb was shorter) was recorded after operation and confirmed at the follow-up in nine cases (numbers 1, 2, 4, 6, 7, 9, 10, 13, 15), and in two cases, the affected limb was longer (numbers 19 and 22). There were no statistically significant differences between groups (*p* = 0.08) (Table 2).

**Table 2:** Clinical and radiological data

Case	LLn post-op	LLn FU-12m	LLp post-op	LLp FU-12m	Neck-n post-op	Neck-n FU-12m	Neck-p post-op	Neck-p FU-12m	Flex–n FU-12m	Flex-p FU-12m	Inter-n FU-12m	Inter-p FU-12m	Exter-n FU-12m	Exter-p FU-12m
1	74.00	78.00	73.00	77.50	76.15	80.10	75.30	79.80	110°	110°	40°	35°	45°	45°
2	69.00	72.50	68.50	72.00	64.73	69.34	63.13	68.45	110°	110°	30°	30°	40°	40°
3	78.00	83.00	78.00	83.00	84.73	90.12	83.96	89.32	120°	120°	35°	35°	45°	45°
4	72.50	76.50	72.00	76.00	76.81	79.71	77.73	80.95	110°	100°	35°	35°	45°	45°
5	68.50	71.00	68.50	71.00	65.80	69.02	64.73	68.80	120°	120°	30°	30°	45°	45°
6	69.00	74.00	68.00	73.00	68.54	72.00	67.45	71.05	115°	115°	30°	30°	50°	45°
7	75.00	79.00	74.50	79.00	77.54	81.49	80.70	85.09	115°	115°	35°	35°	55°	55°
8	72.00	78.00	72.00	78.00	77.83	81.13	76.54	80.76	120°	120°	35°	35°	50°	50°
9	77.50	81.50	77.00	82.00	85.65	90.70	83.95	89.12	115°	115°	30°	30°	45°	45°
10	71.00	74.50	70.50	74.00	66.32	69.80	66.12	69.54	110°	110°	40°	40°	50°	50°
11	72.00	76.50	72.00	76.50	75.87	78.94	74.98	78.04	120°	120°	35°	35°	45°	45°
12	77.50	82.00	77.50	81.50	84.32	89.67	83.22	88.43	115°	115°	30°	30°	45°	45°
13	69.00	72.50	68.00	72.00	67.66	71.23	65.88	70.45	110°	110°	35°	35°	45°	40°
14	75.00	80.00	75.00	80.00	79.57	85.01	78.12	84.22	110°	110°	30°	30°	40°	40°
15	82.00	85.50	81.00	84.50	85.08	91.18	85.01	90.88	120°	120°	35°	35°	55°	50°
16	81.00	85.00	81.00	85.00	84.68	89.12	83.97	88.56	100°	100°	35°	35°	45°	45°
17	73.00	78.00	73.00	78.00	76.90	81.20	75.87	80.64	120°	120°	30°	30°	40°	40°
18	80.00	84.00	80.50	84.00	83.78	89.47	83.12	88.89	115°	115°	30°	30°	40°	40°
19	79.00	84.00	80.00	84.50	84.02	90.02	82.90	88.76	115°	115°	35°	35°	45°	40°
20	69.00	74.50	69.00	74.50	66.78	71.23	65.81	70.75	120°	120°	35°	35°	45°	45°
21	77.00	81.50	77.00	81.50	84.76	89.86	83.82	88.45	115°	115°	30°	30°	45°	45°
22	75.00	80.00	75.50	80.50	86.21	92.03	84.53	89.90	110°	110°	30°	30°	40°	40°
23	68.00	74.00	68.00	74.00	67.21	72.04	66.32	71.84	120°	120°	25°	25°	45°	45
24	71.00	77.00	71.00	77.00	69.05	73.12	68.43	72.53	115°	115°	25°	25°	45°	45°

*LL* limb length (cm), *n* normal unaffected side, *FU* follow-up, *m* months, *p* pathologic affected side, *Neck* length of the neck (mm), *Flex* flexion of the hip, *Inter* internal rotation of the hip, *Exter* external rotation of the hip

As growth of the femoral neck progressed, the screw advanced into the femoral neck from the lateral femoral cortex, closing the gap between the lateral femoral cortex and the screw head (Figs. 1 and 2).

Neck length after operation showed statistically significant differences between the affected and unaffected sides (*p* = 0.001) with a mean difference of 0.7 mm, a coefficient of correlation of 0.99, and a *p* value < 0.0001. At the 12-month follow-up, neck length showed statistically significant differences between the affected and non-affected sides (*p* = 0.02) with a mean difference of 0.5 mm, a coefficient of correlation of 0.99, and a *p* value < 0.0001. Note the statistically significant differences between *T* = 0 and *T* = 12 months (*p* < 0.001). This data indicates that the neck grew in the same way on the affected and unaffected sides during the first-year post-treatment (Table 2 and Figs. 1 and 2).

### Clinical outcome

The clinical results in all patients were rated as excellent and all had resumed full activity at the 6th- and 12th-month post-operation. Limb length discrepancy of ≤ 1 cm (the affected limb was shorter) was recorded post operation and confirmed at follow-up in nine cases (numbers 1, 2, 4, 6, 7, 9, 10, 13, 15), and in two cases, the affected limb was longer (numbers 19, 22). There were no statistically significant differences between groups (*p* = 0.08) (Table 2).

No statistically significant differences were observed between affected and unaffected hips in flexion (*p* = 0.3), extension (*p* = 0.3), internal rotation (*p* = 0.3), and external rotation (*p* = 0.4) and no clinical signs of impingement were detected (Table 2).

## Discussion

The data from this study confirm that the femoral neck in patients with mild SCFE can grow after epiphyseal stabilization by a modified screw which does not produce compression on the proximal femoral physis [19]. Premature proximal femoral physeal closure was prevented and potential complications of avascular necrosis and chondrolysis avoided. Screw fixation permitted continued growth of the femoral neck and epiphyseal/physeal complex and remodeling of the epiphysis and metaphysis. This resulted in improvement of the α angle during the first year after stabilization of the epiphysis with significant change noted at 6 months. The improvement observed in the α angle permits to obtain a normal value (that means less than 50°).

Femoral-acetabular impingement (FAI) has been recognized as a cause of hip pain, decreased range of movement, and labral and chondral abnormalities [1–3]. Reports of arthroscopic [27, 28] and open [9, 29] evaluation of hips after mild SCFE suggest rates of acetabular cartilage and labral damage between 75 and 100%. Two studies [30, 31] with intermediate follow-up periods of 6.1 to 14.4 years reported hip pain in one third of the patients with slip angles between 0° and 30°. To measure patient activity levels, Tegner and Lysholm knee assessment scores were used, and decreased scores were found in SCFE patients compared to similarly aged counterparts. In mild and moderate slips, computer models [30] demonstrated impaction-type impingement caused by the prominent metaphysis of the neck.

Leunig et al. (2010) [11] believe the ultimate goal of SCFE treatment should be to intervene before irreversible joint injury occurs and that for even mild SCFE, in situ pinning with immediate arthroscopic osteoplasty can reduce or even eliminate hip impingement.

The current standard treatment for mild to moderate SCFE is in situ pinning with stabilization of the slip and premature physeal closure being the primary goals [8]. The premature physeal closure leads to modifications of the epiphyseal–metaphyseal complex which itself becomes a cause of impingement [30, 32]. To avoid this complication, it is necessary to use a type of in situ fixation device which allows continued physeal growth [13, 18–21]. Immediate arthroscopic osteoplasty as Leunig et al. (2010) [11] proposed might not be necessary if the initial treatment is done using such a device.

In patients from our study, the single modified cannulated screw provided stability and allowed improvement of the pathological epiphyseal/physeal and metaphyseal morphology by allowing for continued physeal growth. Reviewing strictly unilaterally involved patients allowed us to compare proximal femoral growth and development in the affected and unaffected sides. At follow-up, radiological measurements demonstrated a statistically significant (*p* = 0.01) prolongation in longitudinal femoral neck growth on the involved side. The degree of alteration of the endochondral ossification that would allow for substantial growth and remodeling is unknown. In our series, once the epiphysis was stabilized by our modified screw fixation method, the physis grew in a similar way as the opposite side (Table 2). At the 12-month follow-up, no physeal closures were present.

The study limitations are as follows: (a) a comparison with standard screw fixation cohorts, as we considered only the results reported in other papers [8, 30, 32]; (b) short follow-up; and (c) the considered cohort only affected by mild slip.

## Conclusions

The modified screw fixation device used in our study provided stability, neck growth, and physeal junction remodeling during treatment for mild SCFE in the first period (positive progress observed as early as the 6-month follow-up). By restoring the physiology of the proximal femoral anatomy, potential femoroacetabular impingement was prevented.

## Abbreviations

AP: Anteroposterior
FAI: Femoro-acetabular impingement
PSA: Posterior sloping angle
SCFE: Slipped capital femoral epiphysis
